# Disparities of Care for African-Americans and Caucasians with Community-Acquired Pneumonia: A Retrospective Cohort Study

**DOI:** 10.1186/1472-6963-10-143

**Published:** 2010-05-27

**Authors:** Christopher R Frei, Eric M Mortensen, Laurel A Copeland, Russell T Attridge, Mary Jo V Pugh, Marcos I Restrepo, Antonio Anzueto, Brandy Nakashima, Michael J Fine

**Affiliations:** 1 The University of Texas at Austin College of Pharmacy, 1 University Station, A1900, Austin, TX 78712, USA; 2 The University of Texas Health Science Center at San Antonio, 7703 Floyd Curl Drive, San Antonio, TX 78229, USA; 3 South Texas Veterans Health Care System, Audie L. Murphy Division, 7400 Merton Minter Blvd, San Antonio, TX 78229, USA; 4 VERDICT Research Program, South Texas Veterans Health Care System, 7400 Merton Minter Blvd, San Antonio, TX 78229, USA; 5 Center for Health Equity Research and Promotion, Veterans Affairs Pittsburgh Healthcare System, 7180 Highland Drive (151C-H), Pittsburgh, PA 15206, USA; 6 Division of General Internal Medicine, Department of Medicine, University of Pittsburgh, 200 Lothrop Street, Pittsburgh, PA 15213, USA

## Abstract

**Background:**

African-Americans admitted to U.S. hospitals with community-acquired pneumonia (CAP) are more likely than Caucasians to experience prolonged hospital length of stay (LOS), possibly due to either differential treatment decisions or patient characteristics.

**Methods:**

We assessed associations between race and outcomes (Intensive Care Unit [ICU] variables, LOS, 30-day mortality) for African-American or Caucasian patients over 65 years hospitalized in the Veterans Health Administration (VHA) with CAP (2002-2007). Patients admitted to the ICU were analyzed separately from those not admitted to the ICU. VHA patients who died within 30 days of discharge were excluded from all LOS analyses. We used chi-square and Fisher's exact statistics to compare dichotomous variables, the Wilcoxon Rank Sum test to compare age by race, and Cox Proportional Hazards Regression to analyze hospital LOS. We used separate generalized linear mixed-effect models, with admitting hospital as a random effect, to examine associations between patient race and the receipt of guideline-concordant antibiotics, ICU admission, use of mechanical ventilation, use of vasopressors, LOS, and 30-day mortality. We defined statistical significance as a two-tailed p ≤ 0.0001.

**Results:**

Of 40,878 patients, African-Americans (n = 4,936) were less likely to be married and more likely to have a substance use disorder, neoplastic disease, renal disease, or diabetes compared to Caucasians. African-Americans and Caucasians were equally likely to receive guideline-concordant antibiotics (92% versus 93%, adjusted OR = 0.99; 95% CI = 0.81 to 1.20) and experienced similar 30-day mortality when treated in medical wards (adjusted OR = 0.98; 95% CI = 0.87 to 1.10). African-Americans had a shorter adjusted hospital LOS (adjusted HR = 0.95; 95% CI = 0.92 to 0.98). When admitted to the ICU, African Americans were as likely as Caucasians to receive guideline-concordant antibiotics (76% versus 78%, adjusted OR = 0.99; 95% CI = 0.81 to 1.20), but experienced lower 30-day mortality (adjusted OR = 0.82; 95% CI = 0.68 to 0.99) and shorter hospital LOS (adjusted HR = 0.84; 95% CI = 0.76 to 0.93).

**Conclusions:**

Elderly African-American CAP patients experienced a survival advantage (i.e., lower 30-day mortality) in the ICU compared to Caucasians and shorter hospital LOS in both medical wards and ICUs, after adjusting for numerous baseline differences in patient characteristics. There were no racial differences in receipt of guideline-concordant antibiotic therapies.

## Background

Prior studies have documented health disparities between African-Americans and Caucasians who present to U.S. hospitals with common infectious diseases [1-4]. Health policies, research initiatives, and targeted educational campaigns have sought to bridge this quality chasm [5-7].

In the treatment of community-acquired pneumonia (CAP), several initial processes of care have been associated with improved survival, including the timely delivery and appropriate selection of antibiotic therapy, and acquisition of blood cultures prior to initiating antibiotic therapy [8-11]. These processes have been recommended by professional medical societies, clinical practice guidelines, hospital accreditation commissions, and federal agencies [12]; however, whether such processes of care are performed in an equitable manner for patients of all races is unclear. While some studies have demonstrated African-American patients are less likely to receive timely initiation of antibiotic therapy, diagnostic bronchoscopy, smoking cessation counseling, and pneumococcal and influenza vaccinations [1,3,13,14], the available evidence suggests no difference between African-Americans and Caucasians in the likelihood of receiving guideline-concordant antibiotic therapy, blood cultures, or assessment of arterial oxygenation [3,14].

Despite the observed racial disparities in several evidence-based processes of care for CAP, most Veteran and civilian studies to date have found equal or lower adjusted mortality rates for African-Americans compared to Caucasians [15-18], with few exceptions [14]. Other evidence suggests African-Americans admitted to U.S. hospitals with CAP are more likely to experience prolonged hospital stays [13,19]. It is unclear whether differences in processes of care, hospital length of stay (LOS) and mortality are independently associated with race or due to differences in baseline patient characteristics that vary by race.

The Veterans Health Administration (VHA), the largest vertically integrated healthcare system in the United States, has been at the forefront of a national quality movement to provide high quality and equitable healthcare for all VHA patients. Therefore, we sought to determine whether there were racial differences in processes of care, hospital LOS, or 30-day mortality for African-American and Caucasian VHA patients with CAP, after adjusting for baseline differences in patient characteristics.

## Methods

This retrospective, cohort study of hospitalized patients with CAP is a secondary analysis of data collected as part of a project to assess the association of treatment with statins on clinical outcomes (e.g., mortality) of patients hospitalized with CAP and/or sepsis in the Department of Veterans Affairs Health Care System (National Institutes of Health/National Institute of Nursing Research Grant Number R01NR010828). We used administrative data from the VHA to examine trends in pneumonia care and mortality. The VHA databases are repositories of clinical data from more than 150 VHA hospitals and 850 VHA clinics [20]. The Institutional Review Board of the University of Texas Health Science Center at San Antonio and the South Texas Veterans Health Care System Research and Development committee approved this study.

### Patient Eligibility

We included VHA patients 65 years and older with a diagnosis of pneumonia in fiscal years 2002-2007. We used previously validated ICD-9 codes (480.0-483.99 or 485-487) for pneumonia to identify potential cases [21]. We excluded patients with a history of HIV/AIDS because these patients might be immunocompromised. We excluded those patients admitted to the hospital or a nursing home within 90 days prior to the CAP admission because these would be considered to be health care associated cases. In addition, we excluded those without antibiotic therapy within 48 hours of admission because these might have had hospital acquired pneumonia. Finally, we excluded those patients with non-veteran status or of races other than African-American or Caucasian.

### Baseline Characteristics

We recorded the patient's age, sex, and marital status (married versus unmarried) at the time of admission. We used VHA Priority Group (PG) as a surrogate for socioeconomic status and disease severity, a previously validated approach [22-24]. Priority Group is used within the VHA to determine a Veteran's eligibility for health benefits and exemption from copayments for pharmacy and health care. When assigning the Veterans to these Priority Groups, the VHA takes into consideration the veteran's physical and mental health status, service history (e.g., Prisoner of War or Purple Heart recipient), and how related the Veteran's illness is to their military service (i.e., "service-connected" disability). Priority Group 1 veterans are exempt from all copayments; Priority Group 5 veterans are eligible for care based on their low income. VHA Priority Group 5 is specifically related to very low income, but a veteran with a higher priority (lower PG, i.e., 1-4) could also be low income. In fact, this is often the case. Priority Group 1 veterans are 50%-100% disabled by a service-connected condition, and thus may be unable to earn a good living (if physically disabled) or form supportive social contacts (if psychiatrically disabled). Catastrophic disablement (PG 4) is independently associated with low income in the U.S. veteran population.

We defined 19 comorbid illnesses using ICD-9 codes in accordance with the Charlson comorbidity scoring system [25], using ICD-9 codes from prior outpatient and inpatient care [26]. We defined alcohol abuse or dependence (ICD-9 codes 291, 303, and 305.0) and drug abuse or dependence (codes 292, 304, 305.2, and 305.9) [27]. Because lung disease is correlated with smoking status, we incorporated several approaches to a smoking indicator, based on prior research with VHA data [28]. Smoking status recorded in the electronic medical record is not included in administrative data extracts, therefore we created an indicator that was true whenever any of the following was found: (a) diagnosis of nicotine dependence (ICD-9 codes 305.1, V15.82), (b) outpatient prescription for smoking cessation products (Zyban, Chantix, varenicline, Nicotrol, nicotine replacement), (c) outpatient visit to smoking cessation clinic, (d) CPT treatment code for smoking cessation (99406, 99407). This measure is expected to undercount patients with a smoking history. VHA enrollees smoke more than the US population per age- and sex-adjusted rates [29], but smoking rates also drop with age and deteriorating health. We also defined organ failure (single or multiple) and sepsis based upon the presence of one or more previously established ICD-9 codes for these conditions [6,7].

Missing data on VHA measures of health services utilization is rare in the administrative data extracts of the electronic medical record system. However, while VHA race data have been validated especially for Caucasian and African-American patients, [30,31] missing data on race/ethnicity is a persistent problem. In VHA databases through 2002 (inpatient data) or 2003 (outpatient data), race/ethnicity was recorded by a clinical or clerical observer in inpatient records and was rarely missing, while outpatient records were characterized by higher rates of missing data. A change in policy was then effected to capture self-reported race, leading to high rates of missing data (up to 60% initially). To improve race/ethnicity data, we used all available years of data. Missing-on-race has been associated with increased survival in VHA cohorts defined by outpatient use [32], no doubt reflecting healthy users' lack of inpatient services where race was more likely to be recorded.

### Antibiotic Prescribing and Other Pneumonia Processes of Care

We evaluated antibiotics received within the first 48 hours of hospital admission per joint American Thoracic Society (ATS) and Infectious Diseases Society of America (IDSA) CAP medical practice guidelines [12]. We defined the following antibiotics as guideline-concordant for medical ward patients: 1) β-lactam plus doxycycline, 2) β-lactam plus macrolide, or 3) antipneumococcal fluoroquinolone. We defined guideline-concordant for patients admitted to the intensive care unit (ICU) as: 1) β-lactam plus macrolide, 2) β-lactam plus antipneumococcal fluoroquinolone, 3) antipneumococcal fluoroquinolone plus clindamycin, 4) antipneumococcal fluoroquinolone plus vancomycin, or 5) antipneumococcal fluoroquinolone plus aminoglycoside. β-lactams included ampicillin, ampicillin-sulbactam, cefepime, cefotaxime, cefpodoxime, ceftazidime, ceftriaxone, ertapenem, imipenem-cilastatin, meropenem, piperacillin-tazobactam, and ticarcillin. Antipneumococcal fluoroquinolones included ciprofloxacin, gatifloxacin, gemifloxacin, grepafloxacin, levofloxacin, moxifloxacin, sparfloxacin, and trovafloxacin. Macrolides included azithromycin, clarithromycin, and erythromycin. Any of these guideline-concordant therapies could have had adjunctive linezolid or vancomycin. These antibiotic recommendations for ward and ICU patients have remained consistent through several versions of the guidelines, including those published by IDSA in 1998, 2003, and 2007 and those published by ATS in 1995 and 2007. Thus, no matter which version of the guidelines was followed, these patients managed between 2002 and 2007 should have received the therapies in question.

Additional processes of care included ICU admission, vasopressor use, and mechanical ventilation. We defined mechanical ventilation using ICD-9A code 96.7, recorded during an ICU stay. We defined vasopressor and inotrope use based upon the receipt of any of the following medications during the hospital stay: dobutamine, dopamine, epinephrine, isoproterenol, metaraminol, norepinephrine, phenylephrine, or vasopressin.

### Length of Hospital Stay and 30-Day Mortality

We abstracted the admission and discharge dates for each stay, then defined hospital LOS as the date of discharge minus the date of admission plus one day for the overall hospitalization. The VHA vital status file provided date of death to assess 30-day mortality. The 30-day mortality metric includes those patients who died in the hospital as well as those who died outside the hospital. We chose this follow-up duration because previous research demonstrated that 30-day mortality is primarily due to underlying illness with pneumonia, whereas 90-day mortality is generally attributable to other comorbid conditions [33]. Previous research has demonstrated that this methodology has a sensitivity of approximately 98% for detecting VHA patients' deaths and is as accurate as the National Death Index [34]. In fact, National Death Index data were incorporated into the VHA's ascertainment of death [34].

### Statistical analysis

We conducted all analyses using JMP 7.0, SAS 9.2 (SAS Corp., Cary, NC), and Stata 10 (StataCorp, College Station, TX). Due to the large sample size, we defined statistical significance as a two-tailed p ≤ 0.0001. Bivariate statistics (chi-square and Student's t-test) compared patient demographics, comorbid conditions, processes of care, LOS, and 30-day mortality for African-American and Caucasian patients with CAP. When assessing study endpoints, patients admitted to the ICU were analyzed separately from those not admitted to the ICU. VHA patients who died within 30 days of discharge were excluded from all LOS analyses. This was done because patients who died rapidly experienced abbreviated lengths of hospital stay. In other words, the length of stay for those very severe patients is artificially short because the patients expired before they could complete an entire episode of care. This approach (i.e., censoring those patients who died from the LOS analyses) is consistent with numerous other peer reviewed publications [35-38].

We used chi-square and Fisher's exact statistics to compare dichotomous variables, the Wilcoxon Rank Sum test to compare age by race, and Cox Proportional Hazards Regression to analyze hospital LOS. We used separate generalized linear mixed-effect models, with admitting hospital as a random effect, to examine associations between patient race and the receipt of guideline-concordant antibiotics, ICU admission, use of mechanical ventilation, use of vasopressors, hospital LOS, and 30-day mortality. Hazard ratios, odds ratios, and 95% confidence intervals were calculated from the generalized linear mixed-effects models. Covariates included all 18 variables listed in Table 1 plus a hospital-level variable. Hazard ratios were reported for hospital LOS; whereas, odds ratios were reported for everything else. For LOS, hazard ratios <1 indicate shorter lengths of stay; whereas, hazard ratios >1 indicate longer lengths of stay.

**Table 1:** Comparison of baseline patient characteristics for African-American and Caucasian patients admitted to VHA Hospitals with CAP (n = 40,878)*

	Total	African-Americans	Caucasians	
Patient Characteristics	(n = 40,878)	(n = 4,936)	(n = 35,942)	P-value
Demographics				
Age (yrs), median (interquartile range)	78 (72-83)	77 (72-82)	78 (72-83)	0.005
Male, %	98%	99%	98%	<0.0001
Married, %	53%	43%	55%	<0.0001
Priority group (PG), %				<0.0001
PG 1	18%	13%	19%	--
PG 2	5%	4%	5%	--
PG 3	9%	7%	9%	--
PG 4	15%	21%	14%	--
PG 5	43%	48%	43%	--
PG 6	<1%	<1%	<1%	--
PG 7	3%	2%	3%	--
PG 8	6%	4%	6%	--
Comorbid conditions, %				
Myocardial infarction	5%	4%	5%	<0.0001
Heart failure	22%	19%	22%	<0.0001
Cerebrovascular disease	16%	16%	16%	0.2
Chronic obstructive pulmonary disease	51%	39%	53%	<0.0001
Liver disease	<1%	<1%	1%	0.2
Diabetes	30%	32%	30%	<0.0001
Renal disease	10%	16%	10%	<0.0001
Neoplastic disease	21%	24%	20%	<0.0001
Tobacco use	37%	32%	37%	<0.0001
Alcohol abuse or dependence	3%	4%	3%	0.0002
Substance abuse or dependence	3%	4%	3%	<0.0001
Organ failure and sepsis, %^†^				
Any organ failure	25%	27%	25%	0.0014
Multiple organ failure	6%	6%	5%	0.0061
Sepsis	4%	5%	4%	0.0009

*These results reflect the bivariate statistical tests (chi-square and Wilcoxon Rank Sum tests).

^†^These variables were coded during hospitalization, but were not necessarily known at admission; therefore, they could represent baseline disease severity or complications.

The 18 variables listed in Table 1 were considered *a priori *to be clinically important, and we included them as covariates in each of the multivariable models. The bulk of these variables were chosen because they were key components of well-validated scoring systems that predict patient mortality among pneumonia patients (e.g., age, sex, heart failure, cerebrovascular disease, liver disease, renal disease, neoplastic disease) [39,40]. Other variables were included because we believed they might impact either patient mortality or hospital length of stay (e.g., marital status, priority group [a surrogate for socioeconomic status], chronic obstructive pulmonary disease, tobacco use, alcohol abuse or dependence, substance abuse or dependence, any organ failure, multiple organ failure, or sepsis).

## Results

Overall, 4,936 African-American and 35,942 Caucasian VHA patients met all eligibility criteria (n = 40,878). The study population had a median (interquartile range) age of 78 (72-83) years; 98% were male and 53% were married. History of tobacco dependence (37%), chronic obstructive pulmonary disease (51%), diabetes (30%), heart failure (22%), neoplastic disease (21%), and cerebrovascular disease (16%) were common. One-quarter (25%) had organ failure while 4% had sepsis, and 12% were admitted to the ICU at some point during hospitalization.

### Baseline Characteristics by Patient Race

As shown in Table 1, compared to Caucasians, a greater proportion of African-American VHA patients were single (57% versus 45%), had a higher prevalence of substance abuse (4% versus 3%), neoplastic disease (24% versus 20%), renal disease (16% versus 10%), and diabetes (32% versus 30%). In contrast, Caucasian VHA patients were more likely to have a history of myocardial infarction (5% versus 4%), heart failure (22% versus 19%), and chronic obstructive pulmonary disease (53% versus 39%). Socioeconomic status, as measured by priority group, also differed significantly between the two cohorts.

### Antibiotic Prescribing and Intensive Care Measures by Race

#### Medical Wards

**Figure 1:**
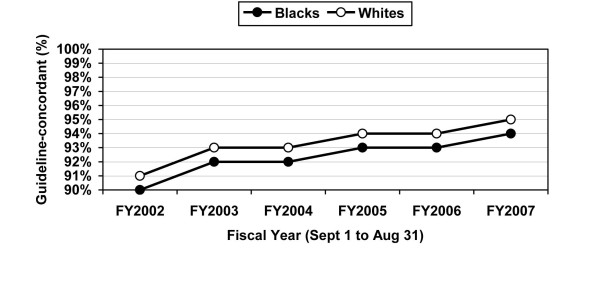
Annual guideline-concordant prescribing for African-American and Caucasian VHA patients with CAP managed in the medical wards (non-ICU patients)*. *The numbers in the figure represent the crude (unadjusted) guideline-concordant antibiotic prescribing rates.

Among VHA patients managed in the medical wards, African-American and Caucasian patients were equally likely to receive guideline-concordant antibiotic therapy in bivariate (92% versus 93%) and multivariable (adjusted OR = 0.99; 95% CI = 0.81 to 1.20) analyses (Tables 2 and 3). Guideline-concordant antibiotic prescribing rates increased over time, from 90-91% in 2002 to 94-95% in 2007 (Figure 1). This possibly reflects greater awareness and appreciation for the benefits associated with the CAP guidelines in the ward setting.

**Table 2:** Antibiotic prescribing, Hospital LOS, and 30-day mortality for African-American and Caucasian VHA patients with CAP managed in the medical wards (non-ICU patients) (n = 35,706)*

	Total	African-Americans	Caucasians	P-value
Guideline-concordant antibiotics^†^	93%	92%	93%	0.0221
LOS, median (interquartile range)^‡^	4 (3-7)	4 (3-7)	4 (3-7)	0.0015
30-day mortality, %	8%	9%	8%	0.0563

LOS = length of stay.

* These results reflect the bivariate statistical tests (chi-square and Wilcoxon Rank Sum tests).

^† ^Guideline-concordant antibiotics were those consistent with 2007 American Thoracic Society/Infectious Diseases Society of America community-acquired pneumonia guidelines[12]. See study methods for more information.

^‡ ^VHA patients who died within 30 days of hospital discharge were excluded from all LOS analyses.

**Table 3:** Multivariable analyses for antibiotic prescribing, Hospital LOS, and 30-day mortality for African-American and Caucasian VHA patients with CAP managed in the medical wards (non-ICU patients) (n = 35,706)*

Dependent variable	HR/OR (95% CI)
Guideline-concordant antibiotics^†^	0.99, 0.81-1.20
LOS^‡^	0.95, 0.92-0.98
30-day mortality	0.98, 0.87-1.10

LOS = length of stay.

* Hazard ratios, odds ratios, and 95% confidence intervals were calculated from the generalized linear mixed-effects models. Covariates included all 18 variables listed in Table 1 plus a hospital-level variable. Hazard ratios were reported for hospital LOS; whereas, odds ratios were reported for everything else. For LOS, hazard ratios <1 indicate shorter lengths of stay; whereas, hazard ratios >1 indicate longer lengths of stay.

^† ^Guideline-concordant antibiotics were those consistent with 2007 American Thoracic Society/Infectious Diseases Society of America community-acquired pneumonia guidelines[12]. See study methods for more information.

^‡ ^VHA patients who died within 30 days of hospital discharge were excluded from all LOS analyses.

#### Intensive Care Units

**Figure 2:**
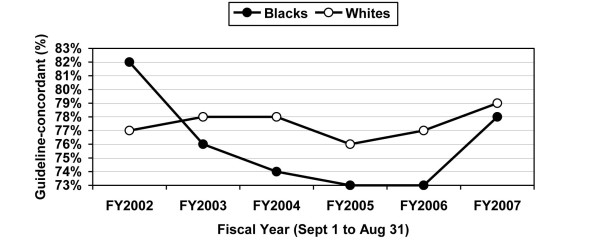
Annual guideline-concordant prescribing for African-American and Caucasian VHA patients with CAP admitted to the intensive care units (ICU patients)*. *The numbers in the figure represent the crude (unadjusted) guideline-concordant antibiotic prescribing rates.

African-American and Caucasian VHA patients experienced similar rates of admission to the ICU (12% versus 13%). Of those patients admitted to the ICU, African-American and Caucasian patients were equally likely to receive guideline-concordant antibiotic therapy in bivariate (76% versus 78%, p = 0.3) and multivariable (adjusted OR = 0.99; 95% CI = 0.81 to 1.20) analyses (Tables 4 and 5). Antibiotic prescribing rates did not differ significantly between Black and White patients admitted to the ICU. Guideline-concordant prescribing was less common in the ICU as compared to the ward, and there was no discernible increase in guideline-concordant prescribing for the latter years studied (Figure 2). This possibly reflects a lack of consensus regarding the most appropriate therapies for CAP patients admitted to the ICU. Use of vasopressors (adjusted OR = 1.27; 95% CI = 1.04 to 1.55) and mechanical ventilation (adjusted OR = 1.40; 95% CI = 1.15 to 1.70) was more common for African-Americans than Caucasians.

**Table 4:** Antibiotic prescribing, intensive care measures, Hospital LOS, and 30-day mortality for African-American and Caucasian VHA patients with CAP admitted to the intensive care units (ICU patients) (n = 5,172)*

	Total	African-Americans	Caucasians	P-value
Guideline-concordant antibiotics^†^	77%	76%	78%	0.3383
Intensive care measures, %				
Intensive care unit admission	13%	12%	13%	0.0023
Vasopressors	26%	32%	26%	0.0005
Mechanical ventilation	42%	48%	41%	0.0005
LOS, median (interquartile range)^‡^	12 (7-23)	13 (7-31)	11 (7-22)	<0.0001
30-day mortality, %	31%	29%	31%	0.2806

LOS = length of stay.

* These results reflect the bivariate statistical tests (chi-square and Wilcoxon Rank Sum tests).

^† ^Guideline-concordant antibiotics were those consistent with 2007 American Thoracic Society/Infectious Diseases Society of America community-acquired pneumonia guidelines[12]. See study methods for more information.

^‡ ^VHA patients who died within 30 days of hospital discharge were excluded from all LOS analyses.

**Table 5:** Multivariable analyses for antibiotic prescribing, intensive care measures, Hospital LOS, and 30-day mortality for African-American and Caucasian VHA patients with CAP admitted to the intensive care units (ICU patients) (n = 5,172)*

Dependent variable	HR/OR (95% CI)
Guideline-concordant antibiotics^†^	0.99, 0.81-1.20
Intensive care measures	
Vasopressors	1.27, 1.04-1.55
Mechanical ventilation	1.40, 1.15-1.70
LOS^‡^	0.84, 0.76-0.93
30-day mortality	0.82, 0.68-0.99

LOS = length of stay.

* Hazard ratios, odds ratios, and 95% confidence intervals were calculated from the generalized linear mixed-effects models. Covariates included all 18 variables listed in Table 1 plus a hospital-level variable. Hazard ratios were reported for hospital LOS; whereas, odds ratios were reported for everything else. For LOS, hazard ratios <1 indicate shorter lengths of stay; whereas, hazard ratios >1 indicate longer lengths of stay.

^† ^Guideline-concordant antibiotics were those consistent with 2007 American Thoracic Society/Infectious Diseases Society of America community-acquired pneumonia guidelines[12]. See study methods for more information.

^‡ ^VHA patients who died within 30 days of hospital discharge were excluded from all LOS analyses.

### 30-day Mortality and Hospital Length of Stay by Patient Race

#### Medical Wards

African-American and Caucasian patients managed on medical wards experienced similar adjusted 30-day mortality (adjusted OR = 0.98; 95% CI = 0.87 to 1.10). African-American patients had a shorter adjusted hospital LOS (adjusted HR = 0.95; 95% CI = 0.92 to 0.98) (Tables 2 and 3).

#### Intensive Care Units

African-American patients admitted to the VHA ICUs experienced lesser adjusted 30-day mortality (adjusted OR = 0.82; 95% CI = 0.68 to 0.99) and shorter adjusted hospital LOS (adjusted HR = 0.84; 95% CI = 0.76 to 0.93) compared to Caucasian patients admitted to VHA ICUs (Tables 4 and 5).

## Discussion

Our principle finding was that African-Americans admitted to VHA hospitals for CAP experienced similar or lower adjusted 30-day mortality compared to Caucasians admitted to VHA hospitals for CAP. Given the size, scope, and inclusion of important covariates, this study is an important addition to the literature. Our analysis of over 40,000 patients from more than 150 VHA hospitals from across the U.S. is one of the largest of its kind. Our study provides a contemporary assessment of guideline-concordant prescribing in both VHA hospital wards and ICUs. Our analysis incorporates patient and hospital characteristics that could be predictive of patient outcome, including patient medical history, social history, socioeconomic status, disease complications, and treatment facility. In so doing, this study provides one of the most robust assessments of health disparities in the U.S. Veteran and civilian literature. The absence of a disadvantage for African-Americans suggests African-American and Caucasian patients admitted to VHA hospitals have equal access to care and receive inpatient treatment of similar quality. Equal access to care is a central goal of health care, and this study brings merit to the initiatives of clinicians striving to care for all patients with the highest standards.

African-American patients admitted to VHA ICUs experienced a survival advantage (i.e., lower 30-day mortality) compared to Caucasian patients admitted to VHA ICUs. While this survival advantage has been documented in other studies [15-18], there is no clear explanation for it. Possibly, the Caucasian patients admitted to VHA ICUs were more severely ill than the African-American patients admitted to VHA ICUs; however, this seems unlikely given that both cohorts had similar rates of ICU admission and vasopressor use and mechanical ventilation were more common among African-Americans. While our study lacked some of the clinical variables required to apply prognostic scoring tools (e.g., PSI, CURB-65, and APACHE-II) [39,40], we included several key covariates including demographic factors (i.e., age, gender) and all five comorbidities (i.e., neoplastic disease, liver disease, heart failure, cerebrovascular disease, and renal disease) used in the PSI score. Thus, our study was well suited to capture/control for the known effects of comorbid illness on mortality for CAP patients. Nevertheless, it is possible that the difference in mortality was due to some unmeasured comorbidity (i.e., receipt of active chemotherapy, immunosuppressive illness, etc) that may have been more prevalent or more problematic for Caucasian patients admitted to the ICU.

The difference in mortality could also be due to differences in advanced directives between the two cohorts. Caucasian patients admitted to VHA hospitals are more likely to have "do not intubate" or "do not resuscitate" orders on file as compared to African-American patients [41]. If this is indeed true, then patients with advanced directives may receive less aggressive therapy. This may partially explain why the African-American patients in our study had higher rates of vasopressor use and mechanical ventilation than the Caucasian patients.

We acknowledge the growing body of literature on frailty [42-44], and recognize the mortality difference might be attributable to differences in frailty between African-American and Caucasian patients admitted to VHA ICUs. The Caucasian patients could be more frail due to a variety of comorbid conditions (i.e., osteoporosis, weight loss or low BMI, cellulitis or decubitus ulcers, fluid electrolyte imbalances, anemia, age >80, syncope, nursing home residence). Likewise, a growing body of literature suggests the importance of functional status, particularly among nursing home patients admitted with pneumonia [45-51]. Poor functional status may contribute to patient mortality and an increased risk of drug resistant pathogens. Our study did not include any measures of frailty or functional status.

In addition to differences in mortality, Caucasian patients in our study remained hospitalized longer than African-American patients. This was the case for both the ward and ICU subgroups. This finding contradicts previous studies reporting longer stays for African-Americans admitted to U.S. hospitals with CAP [13,19]. The previous findings were observed in civilian hospitals and Bennett et al point out that the correlations reported were almost entirely attributable to differences in insurance status and hospital characteristics between racial groups. We did account for priority group and admitting hospital in our multivariable, generalized linear mixed-effect models. We also accounted for 18 other baseline patient characteristics, including several that were significantly different between the two cohorts: marital status, myocardial infarction, heart failure, chronic obstructive pulmonary disease, diabetes, renal disease, neoplastic disease, tobacco use, and substance abuse. Nevertheless, it is possible that observed differences in hospital LOS were due to other unmeasured variables.

When differences in hospital LOS are observed between two groups, it is important to consider if the group with the shorter hospital LOS experiences any clinical detriment for having been discharged sooner. This could be evaluated by considering hospital readmission rates or mortality post hospital discharge. We analyzed 30-day mortality and found that African-American and Caucasian patients discharged from VHA hospitals experienced similar 30-day mortality; we can therefore conclude that the African-American patients admitted to VHA ICUs did not experience any additional clinical detriment for having been discharged from the hospital sooner than their Caucasian counterparts.

African-American and Caucasian VHA patients in our study were equally likely to receive guideline-concordant antibiotic therapy in ward and ICU settings, a finding consistent with a previous study [3]. This finding is paramount since numerous studies have previously correlated guideline-concordant prescribing with improved patient survival [10,52,53]. The guidelines recommend slightly different antibiotic regimens in ward and ICU settings, so it is not surprising that the guideline adherence rates differ somewhat in these two settings. In addition, the recommendations for ward patients have been validated to a greater extent than the recommendations for ICU patients [38,54].

We did not evaluate individual guideline-concordant therapies, nor did we attempt to correlate certain guideline-concordant therapies with patient race. We recognize that selection from among the various guideline-concordant therapies must be somewhat customized on the basis of patient history, risk factors for multi-drug resistant pathogens, prior antibiotic exposure, and patient comorbidities. National pneumonia guidelines encourage prescribers to consider these things when selecting empiric antibiotic therapies [12]. Since African-American and Caucasian patients differed on several baseline characteristics and comorbidities, we would expect to see slight variations in prescribing within the guideline-concordant therapies. A few of those divergent baseline characteristics are described here along with possible implications for empiric antibiotic therapy.

African-American VHA patients were more likely to have a history of substance abuse or dependence, neoplastic disease, renal disease, and diabetes. In contrast, Caucasian VHA patients were more likely to use tobacco, or have a history of heart failure or chronic obstructive pulmonary disease. These observed differences in comorbidities are consistent with other studies in various infectious diseases, which demonstrate that African-Americans often present to hospitals with more comorbidities compared to Caucasians [3,7,19,55]. Patients with diabetes or renal disease may be less likely to receive fluoroquinolone antibiotics to avoid accumulation in renal disease or a perpetuation of glycemic abnormalities. On the other hand, if patients with renal disease receive antibiotics that are excreted by the kidneys (i.e., fluoroquinolones or β-lactams), then they get greater antibiotic exposure, and possibly better efficacy, from the same dose due to their renal compromise. This may impact hospital LOS as recent data suggest certain guideline-supported therapies are associated with shorter hospital stays [56].

Past studies have demonstrated that African-American patients are less likely to receive timely antibiotic therapy and diagnostic bronchoscopy [1,3,13]. We did not capture or evaluate antibiotic timing in our study. We also did not evaluate bronchoscopy; however, we did quantify and compare use of vasopressors and mechanical ventilation between African-American and Caucasian cohorts. We found that African-Americans were more likely to receive these intensive therapies as compared to Caucasians. We did not evaluate acquisition of blood cultures or oxygenation assessment; two process measures previously found to be similar for African-American and Caucasian VHA patients [3].

Our study has limitations that are inherent to all retrospective cohort studies. The results attributed to one explanatory variable could be the result of some unmeasured variable. Any relationships uncovered cannot be definitively considered causative.

The VHA patients in this study are not necessarily representative of all U.S. patients or even all U.S. veterans [57]. The VHA caters to the most disadvantaged veterans and VHA patients are, on average, poorer and sicker than U.S. residents or U.S. veterans [57]. Those with highly service-connected disabilities also receive entry into the VHA system preferentially, and service-connected disability of 50% or more (VA Priority Group 1) garners care without copayments. Lower priority groups (PG 1-6) generally incur copayments for medication prescription, and the lowest priority VA patients (PG 7-8) also have copayments for care. Also, it is certainly possible that African-Americans managed in the VHA system are different from African-Americans managed in other systems.

Additionally, this study was potentially limited by the fact that the investigators were unable to fully exclude VHA patients with healthcare-associated pneumonia (HCAP); however, the stringent exclusion criteria should have minimized these patients. For instance, VHA patients admitted to the hospital or a nursing home within the 90 days prior to the index admission for CAP were excluded. Prior studies have demonstrated that these two exclusions account for a large proportion of HCAP patients [58,59]. As with any study with non-prospective data collection, we are unable to fully exclude the possibility of missing data. However, VA data have been demonstrated to have excellent validity and reliability [60]. Finally, while errors occur in all databases, as well as in chart data, the VHA databases have been shown to be extraordinarily valid with respect to coding of diagnoses especially on inpatient encounters [60].

## Conclusions

Elderly African-American CAP patients experienced a survival advantage (i.e., lower 30-day mortality) in the ICU compared to Caucasians and shorter hospital LOS in both medical wards and ICUs, after adjusting for numerous baseline differences in patient characteristics. There were no racial differences in receipt of guideline-concordant antibiotic therapies.

## Competing interests

The authors declare that they have no competing interests.

## Authors' contributions

CRF participated in the design, data analysis and interpretation, performed statistical analyses, and was the primary person responsible for drafting the manuscript. EMM conceived and designed the primary study; and participated in the design, data analysis and interpretation, performed statistical analyses, and revised the manuscript for important intellectual content. MJP participated in the design, data acquisition, and manuscript revisions. RTA participated in data analysis and interpretation and was the secondary person responsible for drafting the manuscript. MJP and LAC participated in the design, data acquisition, and manuscript revisions. MIR, AA, BN, and MJF participated in the design and manuscript revisions. All authors read and approved the final manuscript.

## Pre-publication history

The pre-publication history for this paper can be accessed here:

http://www.biomedcentral.com/1472-6963/10/143/prepub
